# Environmental Heterogeneity Drives Distinct Spatial Distribution Patterns of Microbial Co-Occurring Species Across Different Grassland Types

**DOI:** 10.3390/microorganisms14010156

**Published:** 2026-01-10

**Authors:** Wenjing Liu, Kai Xue, Biao Zhang, Shutong Zhou, Weiwei Cao, Kui Wang, Yanbin Hao, Xiaoyong Cui, Yanfen Wang

**Affiliations:** 1 College of Resources and Environment, University of Chinese Academy of Sciences, Beijing 100049, China; liuwenjing18@mails.ucas.ac.cn (W.L.);; 2 Beijing Yanshan Earth Critical Zone National Research Station, University of Chinese Academy of Sciences, Beijing 101408, China; 3 State Key Laboratory of Earth System Numerical Modeling and Application, University of Chinese Academy of Sciences, Beijing 100049, China; 4 The College of Life Science, Northwest University, Xi’an 710127, China; 5 College of Life Sciences, University of Chinese Academy of Sciences, Beijing 101408, China

**Keywords:** grassland, soil microbial community, microbial interactions, spatial pattern

## Abstract

Grasslands, as dominant terrestrial ecosystems, significantly influence soil microbial communities through alterations in soil properties. However, their effects on spatial patterns of soil microbial communities are still under-investigated. To address this, we quantified taxa–area (TAR) and node–area (NAR) relationships for prokaryotic and fungal communities across temperate steppe (TS), alpine steppe (AS), and alpine meadow (AM). Our findings indicated that the spatial turnover of both prokaryotic and fungal communities were higher in alpine steppe and alpine meadow than in temperate steppe, mirroring the gradient of soil environmental heterogeneity. Notably, overall species richness increased logarithmically with sampling area in all grasslands; in striking contrast, co-occurring richness exhibited an increasing and then decreasing trend in AS and AM, but declined monotonically in TS, indicating that microbial interaction networks collapse once a critical spatial threshold is exceeded regulated by ecosystem type and environmental heterogeneity. In growing season, the stochastic dominance in prokaryotic assembly (Normalized stochasticity ratio = 0.71–0.89) and deterministic dominance in fungal assembly (Normalized stochasticity ratio = 0.23–0.37) can be explained by their differences in niche breadth and migration rate. These scale-dependent biogeographic patterns demonstrate that grassland type impacts distinct interactions and spatial patterns of microbial communities. These findings provide novel insights into a comprehensive understanding of how grassland type mediates soil microbial community.

## 1. Introduction

Grasslands, covering approximately 20% of the global terrestrial surface [1], provide critical ecosystem services, mainly soil and water conservation [2], climate regulation [3] and habitat for biodiversity. As a key regulator of grassland ecosystems, microorganisms play multiple critical roles in promoting nutrient cycling [4], maintaining soil health and structural stability [5], enhancing ecosystem resilience [6] and regulating plant–soil interactions [7]. Grasslands can be categorized into various types based on environmental gradients. The diversity of grassland types significantly influence soil microbial community composition and diversity through differences in soil physicochemical properties [8,9,10], vegetation composition [11,12,13] and microclimate [14,15]. Exploring the effects of different grassland types on microbial community structure is essential for understanding the mechanisms that maintain the function and stability of grassland ecosystems.

Microorganisms, however, do not operate in isolation. They interact through diverse direct and indirect interactions, such as mutualism, parasitism, predation, and competition [16]. These interactions are important to understand biodiversity maintenance [17] and underpin ecosystem functioning [18]. Network analyses infer potential ecological interactions among microorganisms by calculating statistical correlations between species abundances, revealing keystone microbial taxa [19,20], key ecological functions [21,22,23], and their response mechanisms to environmental disturbances [24,25,26]. Several studies have reported that the complexity of network structure can effectively reflect the community stability and resilience of microbial communities [27,28,29]. For example, highly connected networks enable communities to maintain core ecological processes despite the loss of certain species by providing multiple pathways for interactions [30,31], and significant modularity can reduce disturbances caused by environmental stresses [32,33,34]. But fewer studies focus on the spatial pattern of microbial network structure across different ecosystems.

The taxa–area relationship (TAR), one of the most fundamental and widely recognized patterns in ecology, describes a consistent increase in species richness with expanding sampling area within a defined region [35,36]. Spatial turnover rate can be quantified through the slope of this relationship [37,38]. Numerous studies have documented the spatial patterns of overall microbial community richness across various ecosystems, including the Loess Plateau [39], forest [40], tundra [41,42], glacier [43], desert and typical steppe ecosystems [44]. While the TAR only reveals the accumulation of overall community species richness, ignoring the emergence of key ecological interactions. Nodes in a microbial co-occurrence network reflect the diversity of microorganisms involved in the interaction and can measure the strength of potential interactions between species [22]. By exploring the spatial pattern of the number of network nodes, it can characterize spatial patterns from the perspective of microbial interactions, thereby revealing the underlying mechanisms that maintain biodiversity at both local and regional scales.

It is widely recognized that microbial community assembly is governed by deterministic and stochastic processes, both of which are critical for shaping spatial patterns of microbial community [45,46]. Deterministic processes refer to those in which community composition is shaped by abiotic conditions (e.g., soil pH, temperature, nutrient concentrations) and biotic interactions (e.g., competition, mutualism) [47,48]. In contrast, stochastic processes emphasize that all species are ecologically equivalent, meaning that species diversity and abundance are primarily influenced by random events such as birth, death, dispersal limitation, and random migration, rather than ecological niche differentiation [49]. Their relative importance varies with spatial scales and environmental conditions [50,51,52,53]. Therefore, it is essential to investigate the spatial patterns and community assembly of soil microbial communities across grassland types to clarify the underlying principles that maintain microbial diversity.

While previous microbial geography studies have focused mostly on a single grassland type, comparisons between different grassland types have been less involved, limiting our overall understanding of the spatial distribution patterns of microbial communities in diverse grassland environments. In this study, we implemented a nested sampling design across temperate steppes on the Inner Mongolia Plateau, alpine steppes and alpine meadows on the Qinghai–Tibet Plateau, collecting a total of 182 soil samples. We adopted the taxa–area relationship and node–area relationship (NAR) to analyze spatial patterns of overall and co-occurring microbial richness, respectively. To quantify community assembly mechanisms, the neutral community model (NCM) and normalized stochasticity ratio (NST) were applied. This study aims to: (1) explore the spatial patterns of community richness in both overall and co-occurring microbial communities; (2) compare the spatial turnover rates of microbial communities across grassland types; and (3) clarify the driving factor governing microbial community structure and spatial patterns.

## 2. Materials and Methods

### 2.1. Site Characteristics and Sampling

Based on the differences in vegetation communities and soil types, three grassland types were investigated in this study: alpine steppe (AS), alpine meadow (AM), and temperate steppe (TS). The alpine grasslands (AM and AS) were located in the Nagqu City of Tibet Autonomous Region, China (83°55′–95°5′ E, 29°55′–36°30′ N), with a mean elevation of approximately 4453 m. This region has a typical alpine climate, characterized by cold and arid conditions. The mean annual temperature ranges from −3 °C to 3 °C, and the mean annual precipitation ranges between 200 and 500 mm. Based on the Chinese soil system classification, the soil in AS belongs to Mat-Cryic Cambisol [54], while the soil in AM belongs to the type of chernozem and chestnut [55]. In the AS (Baingoin County), the dominated plant species *Stipa purpurea*, whereas *Kobresia pygmaea* dominates the site in Nagqu City (AM). The TS is situated in Baiyin Xile, Xilingol League, Inner Mongolia (116°67′52″–116°74′79″ E, 43°55′43″–43°59′89″ N), with an elevation range of 1191–1230 m. This region features a typical semi-arid temperate climate, with an average annual precipitation of approximately 350 mm and an average annual temperature of about −0.4 °C. The representative soils of the region are chernozem and kastanozem [56]. The dominant vegetation is *Leymus chinensis*.

The sampling strategy was designed following the nested sampling to overcome the limitations of traditional sampling based on transect intervals (Figure S1). The sample plots at smaller scales are contained within those at larger scales, with sample areas ranging from 0.5 × 0.5 m^2^ to 2048 × 2048 m^2^. Sampling locations were established at the corners of each experimental plot, maintaining a minimum distance of 20 m from the edges of fenced areas. During the growing season (July to August), three representative fenced plots (each with enclosure durations exceeding 10 years) were selected from the Alpine Steppe (AS) and Alpine Meadow (AM) on the Qinghai–Tibet Plateau, and the Temperate Steppe (TS) on the Inner Mongolia Plateau.

After completing aboveground biomass collection for each 0.5 × 0.5 m^2^ sample plot, topsoil was collected from 0 to 10 cm depth using a 7 cm diameter soil auger. Within each quadrat, three randomly positioned soil cores were collected and thoroughly mixed to form a composite sample. Each soil sample was divided into three parts: one stored at −80 °C for molecular analyses, one maintained at 4 °C for biochemical assessments, and one air-dried for subsequent chemical analysis.

### 2.2. DNA Extraction, PCR Amplification and Bioinformation Analyses

DNA was extracted from 0.25 g soil aliquots employing the PowerSoil DNA Isolation Kit (MO BIO Laboratories, Carlsbad, CA, USA). DNA purity and concentration were verified using a NanoDrop-2000 spectrophotometer (Thermo Scientific, Waltham, MA, USA). For prokaryotic community analysis, the V4–V5 regions of the 16S rRNA gene were targeted for amplification using the barcode primer 515F (5′-GTGCCAGCMGCCGCGGTAA-3′) and 907R (5′-CCGTCAATTCMTTTRAGTTT-3′). Fungal communities were characterized through amplification of the ITS2 region with the barcode primer gITS7 (5′-GTGARTCATCGARTCTTTG-3′) and ITS4 (5′-TCCTCCGCTTATTGATATGC-3′). All polymerase chain reaction (PCR) amplifications were conducted following the thermal cycling parameters established in Zhang et al. (2024) [37]. The amplicons obtained were purified and subjected to high-throughput sequencing using an Illumina HiSeq platform at Magigene Biotechnology Co., Ltd. (Guangzhou, China).

The paired-end reads were assembled via FLASH (Fast Length Adjustment of Short reads) following the removal of barcodes and primers [57]. Subsequent bioinformatic processing was performed with USEARCH 8.0, incorporating quality control, length trimming, chimera detection, operational taxonomic unit (OTU) clustering, and taxonomic classification [58]. OTUs were defined at 97% sequence similarity threshold [59]. Taxonomic assignment for prokaryotic communities were conducted against the SILVA 138.1 database [60], while fungal communities were classified using the UNITE database v7.1 [61]. To ensure comparative analysis across samples, sequence data were normalized by resampling to equal number of 707,037 reads for 16S rRNA and 9874 reads for ITS, respectively, using the “resample” package [62] in R v4.1.3.

### 2.3. Soil Physicochemical Properties

Soil water content (SWC) was measured by oven-drying 5 g of fresh soil at 105 °C for 12 h until constant weight was obtained. Soil pH was measured electrometrically with a laboratory pH meter (STARTER3100, Ohaus Instruments Co., Ltd., Shanghai, China) in a 1:2.5 soil-water ratio. Soil organic carbon content (SOC) was quantified with an elemental analyzer (Liqui TOC II; Elementar Analysensysteme GmbH, Hanau, Germany). Total nitrogen concentration (TN) was determined by continuous flow analysis (SEAL Analytical GmbH, Norderstedt, Germany). The quantification of total phosphorus (TP) was performed via UV-VIS spectrophotometry (UV2700, SHIMADZU, Tokyo, Japan). For ammonium nitrogen (NH_4_^+^-N) and nitrate nitrogen (NO_3_^−^-N) extraction, soil samples were mixed with 2 mol/L potassium chloride solution and subjected to 1 h shaking. After centrifugation, the supernatant was analyzed spectrophotometrically at wavelengths of 420 nm and 210 nm for NH_4_^+^-N and NO_3_^−^-N, respectively.

### 2.4. Statistical Analysis

The significant differences in microbial diversity and soil environmental heterogeneity were subjected to one-way analysis of variance (ANOVA), and the means were separated using a least significant difference (LSD) test at 5% probability in the “vegan” R package.

To evaluate soil environmental heterogeneity under different grassland types, we calculated Bray–Curtis dissimilarity using standardized measurements of all measured soil variables (SWC, pH, TOC, TN, TP, NH_4_^+^-N and NO_3_^−^-N). Microbial alpha diversity was assessed through richness, Shannon-Wiener index and Simpson index. Beta diversity was examined using Bray–Curtis dissimilarity matrices generated from OTU abundance data.

The spatial distribution patterns of microbial communities were analyzed using the TAR. The slope of the TAR represents the spatial turnover rate of microbial communities. The TAR was fitted using the “mmSAR” package in R [63], and significant differences in spatial turnover rates among different treatments were assessed with the “simba” package [64].

The richness of co-occurring microbial species was quantified from the number of co-occurrence networks nodes. We introduce the NAR through co-occurrence network analysis, which quantified how the number of network nodes changes with increasing sampling area to uncover variations in microbial interactions across spatial scales. Co-occurrence networks were constructed based on strong and significant Spearman correlations (∣r∣ > 0.80, *p* < 0.05). Before to network construction, OTUs with relative abundances below 0.1% or detected in fewer than 20% of all samples were excluded from the analysis. Topological network properties were extracted and calculated using the “igraph” package in R 4.1.3.

We employed NST analysis to differentiate between stochastic and deterministic processes in microbial community assembly [65]. According to this framework, NST values > 0.5 indicate stochastic process dominance, whereas values < 0.5 suggest deterministic processes govern community assembly. To further evaluate the role of ecological drift and dispersal limitation, we applied the neutral community model (NCM) using the “Hmisc, minpack.lm, and stats4” packages in R [66]. Niche breadth of microbial communities under different treatments was calculated using the “spaa” package in R [67]. Additionally, mantel tests were performed to examine associations between soil environmental factors and microbial community composition using Bray–Curtis dissimilarity matrices. Canonical correspondence analysis (CCA) was performed to examine the relationship between microbial species abundance and soil environmental factors.

All statistical analyses in this study were performed using R version 4.1.3, with the significance level defined as *p* < 0.05.

## 3. Results

### 3.1. Soil Environmental Heterogeneity Across Different Grassland Types

Soil physicochemical properties differed significantly across different grassland types (Table S1, *p* < 0.05). SWC, NH_4_^+^-N, SOC, TN and TP in AM were significantly higher than those in AS and TS, while NO_3_^−^-N in AS was significantly higher than those of other grassland types.

Soil environmental heterogeneity was quantified using Euclidean distance based on soil environmental factors to compare differences across grassland types. The results revealed significant differences in soil environmental heterogeneity among grassland types, with the AS exhibiting the highest soil environmental heterogeneity, followed by the AM, while the TS showed the lowest soil environmental heterogeneity (Figure S2, *p* < 0.05).

### 3.2. Microbial Community Composition and Diversity Across Different Grassland Types

High-throughput sequencing revealed pronounced grassland-specific characteristics in both composition and diversity of soil microbial community. The number of observed prokaryotic OTUs per sample was 70,490 in TS, 68,766 in AS, and 72,853 in AM. In contrast, fungal OTU numbers were 7318, 18,476, and 3826 across these grassland types, respectively. The prokaryotic community was dominated by eight major phyla (relative abundance > 1%), with *Acidobacteria*, *Proteobacteria*, *Thaumarchaeota*, *Actinobacteria*, *Bacteroidetes*, *Firmicutes*, *Chloroflexi*, and *Planctomycetes* together accounting for over 90% of the total sequences (Figure S3a). The relative abundance of *Actinobacteria* in TS reached 31.49%, which was higher than that in the AS (26.28%) and AM (27.35%). The relative abundance of *Proteobacteria* was 22.83% in the AM, significantly higher than that in the TS (18.09%) and AS (18.07%). *Ascomycota*, *Basidiomycota*, and *Mortierellomycota* were the dominant fungal phyla, collectively accounting for >90% of the total fungal community (Figure S3b). The relative abundance of *Ascomycota* was significantly higher in AS (83.44%) than in AM (71.78%) and TS (69.37%). In contrast, *Basidiomycota* reached its highest relative abundance (16.50%) in AM than in AS (5.02%) and TS (8.44%).

For prokaryotes, species richness was significantly higher in the AM (5066 ± 48.53) than the AS (4657.54 ± 22.30) and TS (3940.65 ± 22.35). In contrast, the AS exhibited higher Shannon and Simpson indices than the other two grassland types (*p* < 0.05, Figure S4a). For fungal communities, both alpha diversity indices in the TS were significantly higher than those in the AS and AM (*p* < 0.05, Figure S4b).

Beta-diversity based on Bray–Curtis dissimilarity revealed significant differences in microbial community composition among grassland types. Specifically, prokaryotic communities in the AM exhibited significantly higher beta diversity than those in both the TS and AS (*p* < 0.05, Figure S5a). In contrast, fungal communities in the AS showed significantly higher beta diversity compared with the TS and AM (*p* < 0.05, Figure S5b).

### 3.3. Spatial Turnover of Microbial Communities Across Different Grassland Types

**Figure 1:**
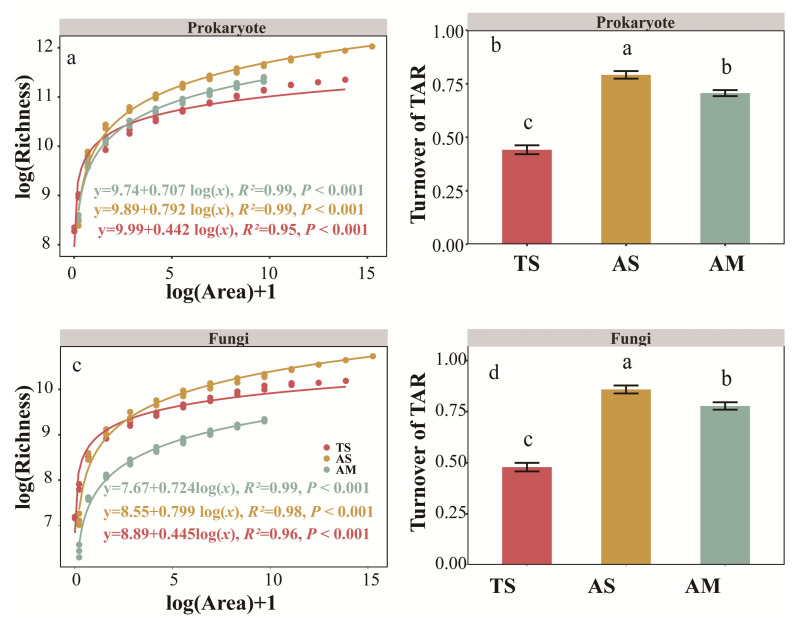
Taxa–area relationship and spatial turnover rate of microbial communities across different grassland types. (**a**,**b**): Prokaryote; (**c**,**d**): Fungi. The spatial turnover rate was calculated from the slope of the taxa–area relationship. Different letters indicate significant differences among grassland types (*p* ≤ 0.05). Values represent mean ± standard error. TS: temperate steppe; AS: alpine steppe; AM: alpine meadow.

The microbial community richness was significantly increased with sampling area for prokaryotes or fungi across different grassland types (*p* < 0.001, Figure 1). For both prokaryotic and fungal communities in the TS, AS and AM, the logarithm model (S = c + z log A) demonstrated a best fit compared to the power model (S = cA^z^). The logarithm model explained nearly all variance (prokaryotes: R^2^ = 0.99; fungi: R^2^ = 0.98–0.99), outperforming the power model (prokaryotes: R^2^ = 0.94–0.95; fungi: R^2^ = 0.81–0.95) (Table S2).

When comparing the slopes of taxa–area relationship across grassland types, turnover rates for both prokaryotic and fungal communities were consistently higher in AS than in AM and TS (AS: prokaryote, 0.792; fungi, 0.799; AM: prokaryote, 0.707; fungi, 0.724; TS: prokaryote, 0.442; fungi, 0.445) (*p* < 0.05, Figure 1).

**Figure 2:**
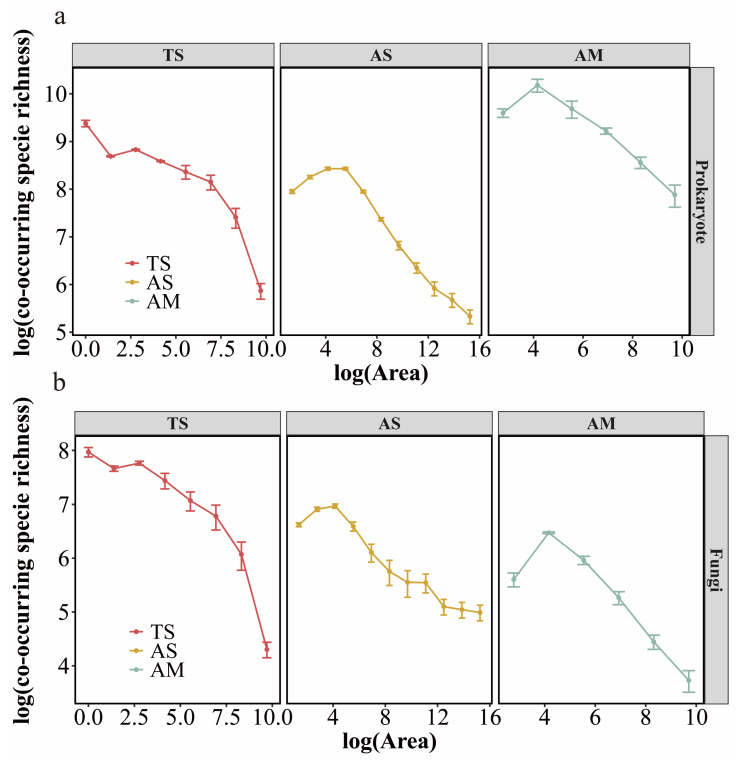
The relationship between the richness of microbial co-occurring species and sampling area across different grassland types (**a**): Prokaryote; (**b**): Fungi. The co-occurring species richness of microbial communities refers to the number of nodes in the co-occurrence network analysis. TS: temperate steppe; AS: alpine steppe; AM: alpine meadow.

In contrast to the TAR, the co-occurring species richness-area relationship exhibited distinct patterns among grassland types for both prokaryotic and fungal communities (Figure 2). In the TS, this relationship showed a declining trend. Conversely, in both the AS and AM, the richness of co-occurring species represented by network node number initially increased with sampling area before reaching a tipping point, beyond which it gradually decreased. For both prokaryotic and fungal communities, the threshold area of the NAR was consistently largest in the AS, followed by the AM and then the TS.

### 3.4. Microbial Community Assembly Across Different Grassland Types

Prokaryotic communities maintained consistently broader niche breadth than fungal communities across all grassland types (Figure S6). Specifically, prokaryotic communities exhibited significantly broader niche breadth in the AS than in the AM and TS (*p* < 0.05, Figure S6a). In contrast, fungi showed significantly greater niche breadth in the TS compared to both alpine grassland types (*p* < 0.05, Figure S6b).

**Figure 3:**
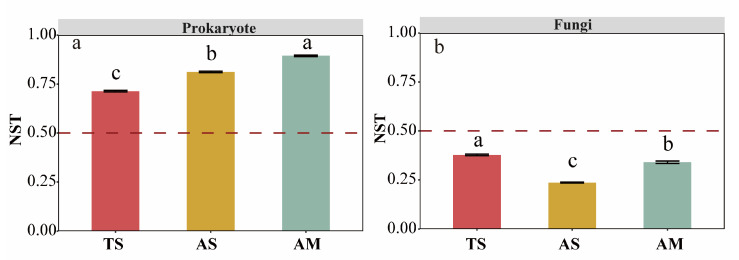
Normalized stochastic ratio of microbial communities across different grassland types (**a**): Prokaryote; (**b**): Fungi. Different letters indicate significant differences among grassland types (*p* ≤ 0.05). Values represent mean ± standard error. Dashed lines represent NST values of 0.5. TS: temperate steppe; AS: alpine steppe; AM: alpine meadow.

The NST index revealed the potentially important roles of both stochastic and deterministic processes in community assembly across grassland types. For prokaryotic communities, stochastic processes (NST = 0.71–0.89) had a more significant influence than deterministic processes (NST = 0.23–0.37) on community assembly. Notably, the impact of stochastic processes was significantly stronger in alpine grasslands (AS: 0.894 ± 0.004; AM: 0.812 ± 0.004) than in the TS (0.714 ± 0.004) (*p* < 0.05, Figure 3a). In contrast, deterministic processes dominated in fungal community assembly, with their influence being significantly greater in the TS (0.378 ± 0.004) than in alpine grasslands (AS: 0.236 ± 0.002; AM: 0.340 ± 0.006) (*p* < 0.05, Figure 3b).

**Figure 4:**
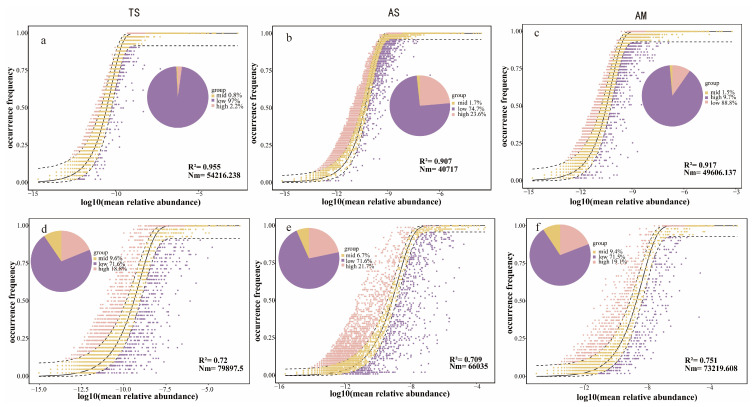
Neutral community model for microbial communities across different grassland types. (**a**–**c**): Prokaryote; (**d**–**f**): Fungi. The solid black line indicates the best-fit curve of the neutral community model, and the dashed black line indicates the 95% confidence interval for the model. OTUs with observed frequencies exceeding the predicted values of the neutral model are marked in pink, and OTUs below the predicted values are marked in purple. The parameter Nm denotes the product of metacommunity size (N) and migration rate (m). TS: temperate steppe; AS: alpine steppe; AM: alpine meadow.

The NCM explained 73.2% of the variance in prokaryotic communities and 50% of the variance in fungal communities (Figure 4), further supporting that prokaryotic communities align more closely with the neutral model than fungal communities. Specifically, the NCM accounted for 91%, 92%, and 95% of the variance in prokaryotic communities in AS, AM, and TS, respectively (Table 1), indicating the dominant role of stochastic processes in prokaryotic community assembly across all sites. The m values for prokaryotic communities were higher in AS (m = 0.93) and AM (m = 0.75) than in TS (m = 0.66), suggesting stronger species dispersal capacity in AS and AM compared to TS.

### 3.5. Environmental Factors Shaping Microbial Community Structure Across Different Grassland Types

The results of Mantel tests (Table 2) revealed that in the TS, prokaryotic community similarity was significantly positively correlated with an increase in the differences between NO_3_^−^-N (r = 0.37, *p* = 0.001), SOC (r = 0.20, *p* < 0.05), and TN (r = 0.20, *p* < 0.01). For fungal communities, community similarity also showed a significant positive correlation with increasing differences in NH_4_^+^-N (r = 0.19, *p* < 0.05), NO_3_^−^-N (r = 0.29, *p* = 0.001), SOC (r = 0.25, *p* < 0.01), and TN (r = 0.25, *p* < 0.01).

In the AS, prokaryotic community similarity was significantly positively correlated with environmental differences in pH (r = 0.08, *p* = 0.05), NH_4_^+^-N (r = 0.16, *p* < 0.01), NO_3_^−^-N (r = 0.22, *p* = 0.001), SOC (r = 0.40, *p* = 0.001), and TN (r = 0.27, *p* < 0.001). In contrast, fungal community similarity in the AS was only significantly correlated with SOC (r = 0.14, *p* = 0.01).

In the AM, prokaryotic community similarity increased significantly with increasing differences in SWC (r = 0.26, *p* = 0.001), pH (r = 0.45, *p* = 0.001), NH_4_^+^-N (r = 0.35, *p* < 0.01), SOC (r = 0.25, *p* = 0.001), TN (r = 0.26, *p* = 0.001), and TP (r = 0.31, *p* = 0.001). However, fungal community similarity in the AM grassland was significantly correlated with all measured environmental factors.

CCA results (Figure S9) demonstrated that SOC was the most influential driver of prokaryotic communities in the TS, accounting for 46.50% of the observed variation. For fungal communities in this grassland type, SWC was the most significant environmental factor, explaining 26.73% of the variation (R^2^ = 26.73%). In the AS, SOC exhibited the strongest correlation with both prokaryotic (R^2^ = 49.99%) and fungal (R^2^ = 41.77%) community structures. In the AM grassland, by contrast, SWC had the highest explanatory for prokaryotic communities (R^2^ = 67.44%), whereas SOC (R^2^ = 41.77%) was identified as the primary factor shaping fungal community composition.

## 4. Discussion

### 4.1. The Spatial Patterns of Microbial Community Across Different Grassland Types

Unlike previous studies, we demonstrated that the TAR for all three grassland types (TS, AS, and AM) best followed the logarithmic model (Figure 2), consistent with our previous work. Zhang et al. (2024) demonstrated that the log-power function provides a superior fit to bacterial taxa–area relationships compared with the traditional power law, effectively reducing the overestimation of soil bacterial diversity at regional scales [37]. For both prokaryotic and fungal communities, the spatial turnover rates were significantly higher in AS and AM than in TS. This variation in spatial turnover can be explained by soil environmental heterogeneity [68]. Our comparative analysis revealed that microbial communities in alpine grasslands not only showed higher spatial turnover rates but also experienced greater soil environmental heterogeneity compared to those in TS (Figure S2). Such heightened heterogeneity likely promoted increased spatial differentiation and diversity in microbial communities, thereby driving faster spatial turnover (Figure S8).

We observed that within the sampling area, the NAR (based on network nodes) differed from the TAR (based on microbial OTUs). The richness of co-occurring species reflects specific microbial taxa whose occurrence is interdependent within the community. Furthermore, microbial interactions were predominantly positive across the different grassland types (Figure S7). The results showed that while the overall microbial community richness increased with the sampling area increasing, the richness of species with positive correlation was limited beyond a certain threshold, which led to its decrease.

The NAR exhibits distinct trends across different grassland types. In alpine grasslands, microbial co-occurring species richness showed an increasing and then decreasing trend with increasing sampling area. This is because co-occurrence network analysis is not constructed based on simple co-occurrence counts of species in the same sample, but rather through statistical inference of correlations in species distributions [69]. Thus, even if certain species do not co-occur in localized small-scale samples, they can still be identified as co-occurring nodes in the network as long as they show significant correlation in the large spatial scale. Furthermore, at smaller scales, species accumulation may be the dominant process, which not only facilitates the coexistence of more species but is ultimately manifested as an increase in co-occurring species richness [70]. As the sampling area expands further, differences in soil conditions between samples increase, eventually exceeding critical thresholds. Environmental filtering and dispersal limitation then act together to exclude some species that are able to coexist at small scales, leading to a declining trend in this relationship. In contrast, temperate steppes are relatively stable and experience weaker environmental stress, which may intensify competitive exclusion among microbial taxa for resources. This heightened competition likely promotes species turnover rather than coexistence, thereby further reducing the abundance of co-occurring species [71].

### 4.2. Niche Breadth Dominates Microbial Community Assembly

The spatial turnover of microbial communities is governed by two main processes: ecological niche selection and dispersal limitation [72]. Ecological niche selection drives compositional change through species selection for their preferred environments [73], whereas dispersal limitation enhances spatial community differentiation by hindering the migration of organisms [74]. Our findings indicated that stochastic processes dominated prokaryotic community assembly across grassland types, whereas deterministic processes dominated fungal community assembly. This contrasted with previous studies reporting deterministic processes as the primary drivers of spatial turnover in both prokaryotic and fungal communities along geographical distances [75]. Our results showed that stochastic processes primarily shaped prokaryotic spatial patterns, in contrast to fungi, which were governed by deterministic processes. This implies that different ecosystems may be structured by distinct community assembly mechanisms. To further assess the role of niche selection, we employed neutral community modeling to evaluate the importance of stochastic processes. Results demonstrated a better fit of the neutral model to prokaryotic than fungal communities, consistent with NST analysis and niche breadth comparisons. This indicated weaker dispersal limitation in prokaryotes, likely attributable to their broader niche widths and higher migration rates.

### 4.3. Microbial Community Composition and Diversity Across Different Grassland Types

For prokaryotic communities, the dominant phyla included *Actinobacteria*, *Proteobacteria*, *Thaumarchaeota*, *Acidobacteria*, and *Bacteroidetes* (Figure S2a), which was consistent with previous studies in temperate grasslands of northern China and alpine grasslands [76,77]. *Actinobacteria* was the dominant prokaryotic phylum across all grassland types may be attributed to their remarkable environmental adaptability and resilience under stressful conditions [78]. By efficiently decomposing cellulose and lignin, Actinobacteria drives soil organic matter turnover in grassland ecosystems [79]. Furthermore, we found that the relative abundance of *Proteobacteria* was significantly higher in AM than that in TS, which was consistent with the findings of studies on alpine ecosystems in the semi-arid region of northwestern China [80]. This difference was mainly attributed to the higher soil moisture and relatively rich organic matter conditions in alpine meadows (Table S1). Together, these environmental factors create a favorable habitat for the growth of eutrophic microorganisms, and thus the proliferation and spread of *Proteobacteria* [81,82]. At the same time, the dominance of the *Proteobacteria* phylum, which contains a large number of key functional taxa that drive organic matter decomposition and nitrogen transformation, implies that the potential for microbial-mediated nitrogen cycling may be stronger in alpine meadow. Regarding fungal composition, the relative abundance of *Basidiomycota* was higher in AM than in AS and TS. This pattern may be attributed to the comparatively higher soil organic matter content in AM, which supplies a substantial carbon source and specialized ecological niches for *Basidiomycota* [83]. In addition, *Basidiomycota* often form close ectomycorrhizal symbioses with plants, and this mutualism relationship further contributes to their abundance [84].

We found prokaryotic alpha diversity was significantly higher in the AS than in the AM and TS, supporting the previous observations of increasing diversity with elevation from low to high altitude grasslands [85]. In contrast, for fungal communities, no clear elevational trend in alpha diversity was detected. This was consistent with global analyses of soil fungal diversity along elevation gradients, which have also failed to identify a general elevational pattern in fungal alpha diversity [86]. These results imply that prokaryotes exhibit high physiological and metabolic adaptability, enabling them to thrive in harsh high-altitude environments and display elevated diversity [87]. In comparison, whether at large or local scales, no consistent elevational pattern in fungal alpha diversity has been established, indicating heterogeneous responses of fungal diversity to local environmental variations along elevation gradients [88].

### 4.4. Soil Organic Carbon and Soil Water Content Are the Main Environmental Factors Relating Community Composition

In the TS and AS, soil organic carbon showed the strongest correlation with prokaryotic community composition. This suggests that the importance of SOC as a limiting factor varies across ecosystems, which is consistent with previous studies [89,90,91,92]. In contrast, soil microbial composition in the AM was most strongly correlated with soil water content. Previous studies have shown that SWC, is a major factor influencing microbial community changes in permafrost regions, which is consistent with our results [93,94]. This is primarily due to concentrated summer precipitation and slow soil water evaporation under low temperature conditions, where water availability becomes a critical factor for microbial survival [95].

For fungal community composition, soil water content emerged as the primary driver in the TS. This may be due to the significant spatial heterogeneity of annual precipitation in the TS, making soil water content a key factor influencing vegetation cover and thus indirectly affecting microbial community structure [96]. Simultaneously, soil moisture directly influences microbial distribution patterns, favoring taxa adapted to water stress [96,97]. In the AS and AM, fungal community composition was primarily correlated with soil organic carbon. In these cold high-altitude environments, fungi rely heavily on organic matter as their main carbon source for survival and metabolism [98]. The slow decomposition of soil organic matter under low temperatures provides a stable and sustained nutrient supply for fungal communities [99,100].

This study focuses on the plant growing season, and the results may not be fully representative of microbial dynamics during the non-growing or transitional seasons. Future studies could further reveal the temporal dynamics of microbial communities through continuous sampling across seasons, leading to a more comprehensive understanding of the geographic distribution patterns of grassland microbes.

## 5. Conclusions

This study demonstrated that there are spatial patterns of soil microbial community in the different grasslands and also revealed their underlying mechanisms. The spatial turnover of prokaryotic and fungal communities was significantly higher in alpine grasslands compared to temperate grasslands, a pattern primarily driven by greater soil environmental heterogeneity in alpine ecosystems. Importantly, co-occurring microbial species richness in alpine grasslands exhibited an increasing and then decreasing trend with sampling area, contrasting with the decreasing trend in temperate steppe. These results demonstrated that microbial interactions were strongly scale-dependent and their stability was mediated by ecosystem-specific conditions. Furthermore, in the growing season, prokaryotic communities were predominantly structured by stochastic processes across all grassland types, while fungal communities were mainly governed by deterministic processes. This study provides novel insights into how spatial scaling and distinct assembly mechanisms collectively shape microbial biogeography in contrasting grassland ecosystems.

## Figures and Tables

**Table 1:** Neutral model fit and species migration rates for microbial communities across different grassland types.

		TS	AS	AM
Prokaryote	R^2^	0.95	0.91	0.92
	m	0.66	0.93	0.75
Fungi	R^2^	0.72	0.71	0.75
	m	0.04	0.03	0.03

The R^2^ value reflects the goodness-of-fit of the neutral community model. The higher R^2^, the community assembly process aligns more closely with neutral theory. The parameter m represents the average migration rate of the community, assuming equal dispersal ability across all species. The lower the value of m, the stronger the dispersal limitation of the community, while the higher the value of m, the greater the freedom of species to migrate and the weaker the dispersal limitation. TS: temperate steppe; AS: alpine steppe; AM: alpine meadow.

**Table 2:** Mantel test for correlation between microbial community variability and soil properties based on Bray–Curtis distance.

		TS		AS		AM	
		r	*p*	r	*p*	r	*p*
Prokaryote	SWC	0.064	0.206	0.081	0.062	0.260	0.001 ***
	pH	0.151	0.071	0.081	0.050 *	0.448	0.001 ***
	NH_4_^+^-N	0.103	0.104	0.164	0.008 **	0.345	0.002 ***
	NO_3_^−^-N	0.369	0.001 ***	0.224	0.001 ***	0.021	0.375
	SOC	0.195	0.013 *	0.392	0.001 ***	0.254	0.001 ***
	TN	0.202	0.005 **	0.273	0.001 ***	0.259	0.001 ***
	TP	0.034	0.267	0.032	0.253	0.311	0.001 ***
Fungi	SWC	0.111	0.142	−0.042	0.744	0.323	0.001 ***
	pH	−0.043	0.585	0.072	0.133	0.117	0.042 *
	NH_4_^+^-N	0.190	0.035 *	−0.024	0.574	0.217	0.004 **
	NO_3_^−^-N	0.287	0.001 ***	0.072	0.133	0.184	0.007 **
	SOC	0.245	0.003 **	0.142	0.010 **	0.225	0.001 ***
	TN	0.248	0.002 **	0.054	0.153	0.265	0.001 ***
	TP	0.108	0.153	−0.024	0.582	0.285	0.001 ***

* indicates significant *p*-value < 0.05; ** = significant at *p*-value ≤ 0.01; *** = significant at *p*-value ≤ 0.001.

## Data Availability

The original contributions presented in this study are included in the article/Supplementary Material. Further inquiries can be directed to the corresponding author.

## Supplementary Materials

The following supporting information can be downloaded at: https://www.mdpi.com/article/10.3390/microorganisms14010156/s1, Figure S1: The nested sampling design and landscape photographs in each grassland; Figure S2: The soil environmental heterogeneity based on Euclidean distance across different grassland types; Figure S3: Relative abundance of Prokaryotic (a) and Fungal (b) communities at the phylum level across different grassland types; Figure S4: The alpha diversity of microbial communities across different grassland types; Figure S5: The beta diversity of microbial communities across different grassland types; Figure S6: Niche breadth of microbial communities across different grassland types; Figure S7: Relative share of positive and negative links in co-occurring networks across different grassland types; Figure S8: Relationship between environmental heterogeneity and microbial spatial turnover rate; Figure S9: Canonical correspondence analysis between soil microbial communities and environmental factors across different grassland types; Table S1: Soil chemical properties across different grassland types; Table S2: TAR model fitting of prokaryotic and fungal communities across different grassland types.

## Abbreviations

The following abbreviations are used in this manuscript:

TAR	Taxa–area relationship
NAR	Node–area relationship
NCM	Neutral community model
NST	Normalized stochasticity ratio
AS	Alpine steppe
AM	Alpine meadow
TS	Temperate steppe
FLASH	Fast Length Adjustment of Short reads
OTU	Operational taxonomic unit
SWC	Soil water content
SOC	Soil organic carbon
TN	Total nitrogen concentration
TP	Total phosphorus
NH_4_^+^-N	Ammonium nitrogen
NO_3_^−^-N	Nitrate nitrogen
ANOVA	One-way analysis of variance
LSD	Least significant difference
CCA	Canonical correspondence analysis
