# Supplementary material for: Environmental Heterogeneity Drives Distinct Spatial Distribution Patterns of Microbial Co-Occurring Species Across Different Grassland Types
Source: Microorganisms. 2026 Jan 10;14(1):156. doi: 10.3390/microorganisms14010156 (PMC12844442; doi:10.3390/microorganisms14010156)
Supplement: Supplementary file 1 [file microorganisms-14-00156-s001.zip › Supplementary File.pdf]

Supplementary Online Material for

Environmental heterogeneity drives distinct spatial distribution patterns of microbial co-occurring species across different grassland types

Wenjing Liu[1], Kai Xue[1,2,3], Biao Zhang[1,4], Shutong Zhou[1,5], Weiwei Cao[1], Kui Wang[1], Yanbin Hao[2,6], Xiaoyong Cui[2,6], Yanfen Wang[1,2,3]*

1 College of Resources and Environment, University of Chinese Academy of Sciences, Beijing 100049, China

2 Beijing Yanshan Earth Critical Zone National Research Station, University of Chinese Academy of Sciences, Beijing 101408, China

3 State Key Laboratory of Earth System Numerical Modeling and Application, University of Chinese Academy of Sciences, Beijing 100049, China

4 The College of LifeScience, Northwest University, Xi'an 710127, China

5 School of Geography and Tourism, Qilu Normal University, Jinan 250200, China

6 College of Life Sciences, University of Chinese Academy of Sciences, Beijing 101408, China

*Correspondence: Kai Xue

Email: xuekai@ucas.ac.cn

Table S1. Soil chemical properties across different grassland types

	Site	TS	AS	AM
	Variables	Mean ± SE	Mean ± SE	Mean ± SE
0-10 cm	SWC (%)	11.71±0.03 b	7.87 ± 0.19 c	12.6 ± 0.49 a
	pH	6.61 ± 0.04	6.67 ± 0.08	6.39 ± 0.03
	NH_4^+-N (mg/kg)	2.88 ± 0.22 c	7.89 ± 0.17 b	17.78 ± 1.84 a
	NO_3^+-N (mg/kg)	2.01 ± 0.03 c	11.46 ± 0.82 a	5.94 ± 0.50 b
	SOC (g/Kg)	22.88 ± 0.64 b	7.55 ± 0.34 c	51.04 ± 1.68 a
	TN (g/Kg)	2.13 ± 0.06 b	0.83 ± 0.04 c	4.48 ± 0.14 a
	TP (g/Kg)	0.42 ± 0.02 b	0.27 ± 0.01 c	0.48 ± 0.01 a

Different letters indicate significant differences among grassland types ($P \leq 0.05$). TS: temperate steppe; AS: alpine steppe; AM: alpine meadow.

Table S2. TAR model fitting of prokaryotic and fungal communities across different grassland types

	Model name	Formula	TS		AS		AM	
			AIC	R^2	AIC	R^2	AIC	R^2
Prokaryote	Power	$S = cA^z$	235.67	0.94	251.96	0.94	162.19	0.95
	Logarithm	S =c +zlog(A)	215.95	0.99	188.56	0.99	111.00	0.99
	Negative exponential	S = c(1-exp(-zA))	251.73	0.78	269.29	0.75	175.01	0.79
	Monod	S = (cA)/(z+A)	247.60	0.84	265.51	0.82	170.83	0.87
	Logistic	S = c/(1+exp(-zA+f))	247.79	0.86	266.09	0.84	173.28	0.86
	Rational function	S =(c+Za)(1+Fa)	250.21	0.83	268.33	0.80	200.19	-1.75
Fungi	Power	$S = cA^z$	210.26	0.91	216.33	0.94	125.39	0.95
	Logarithm	S =c +zlog(A)	189.70	0.98	167.27	0.99	78.99	0.99
	Negative exponential	S = c(1-exp(-zA))	220.73	0.79	236.22	0.74	138.34	0.79
	Monod	S = (cA)/(z+A)	216.13	0.85	232.69	0.80	134.21	0.87
	Logistic	S = c/(1+exp(-zA+f))	216.92	0.87	232.34	0.84	136.70	0.86
	Rational function	S =(c+Za)(1+Fa)	220.07	0.83	234.92	0.80	137.21	0.85

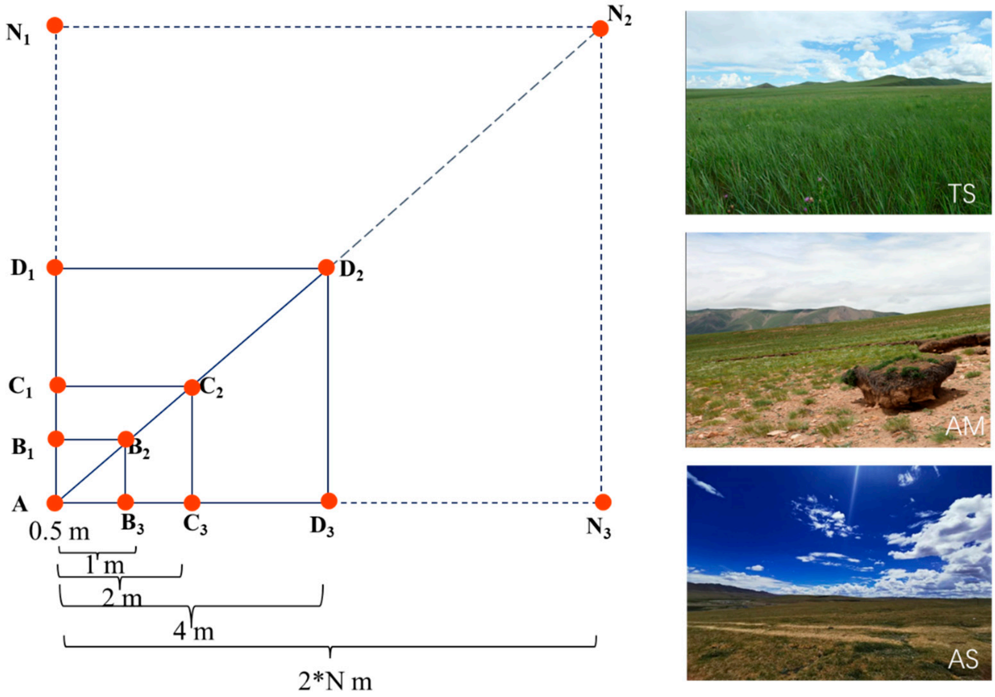

Figure S1. The nested sampling design and landscape photographs in each grassland.

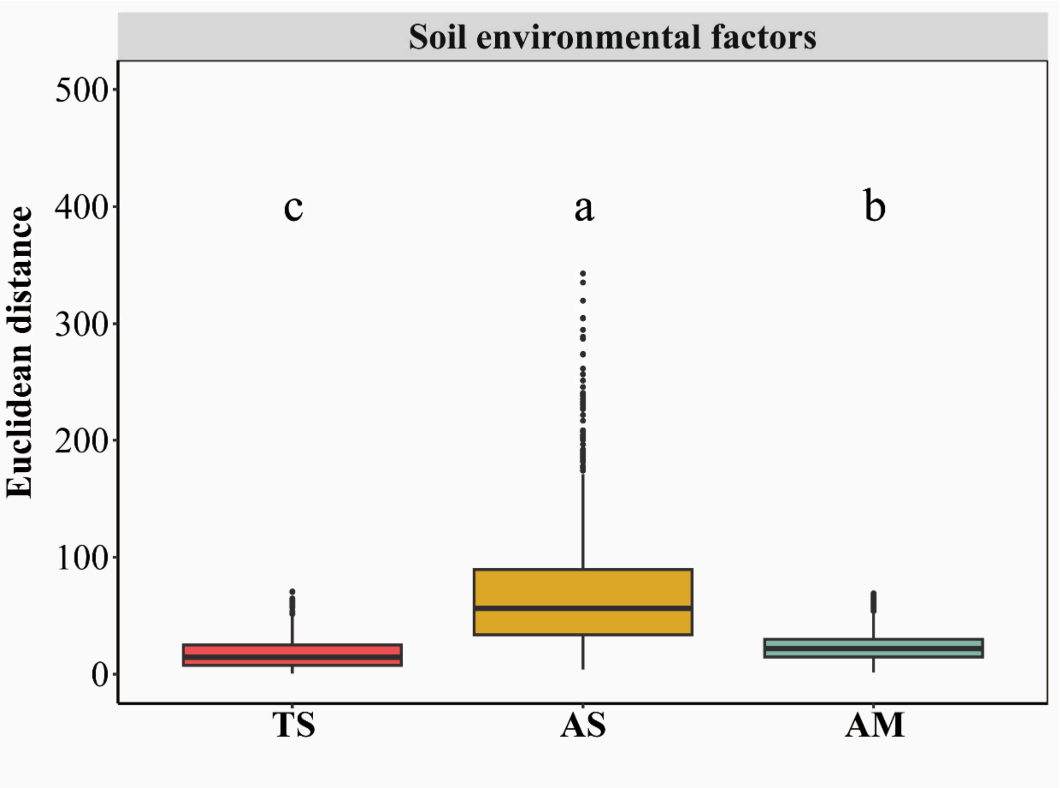

Figure S2. The soil environmental heterogeneity based on Euclidean distance across different grassland types. Different letters indicate significant differences among grassland types ($P \leq 0.05$). TS: temperate steppe; AS: alpine steppe; AM: alpine meadow.

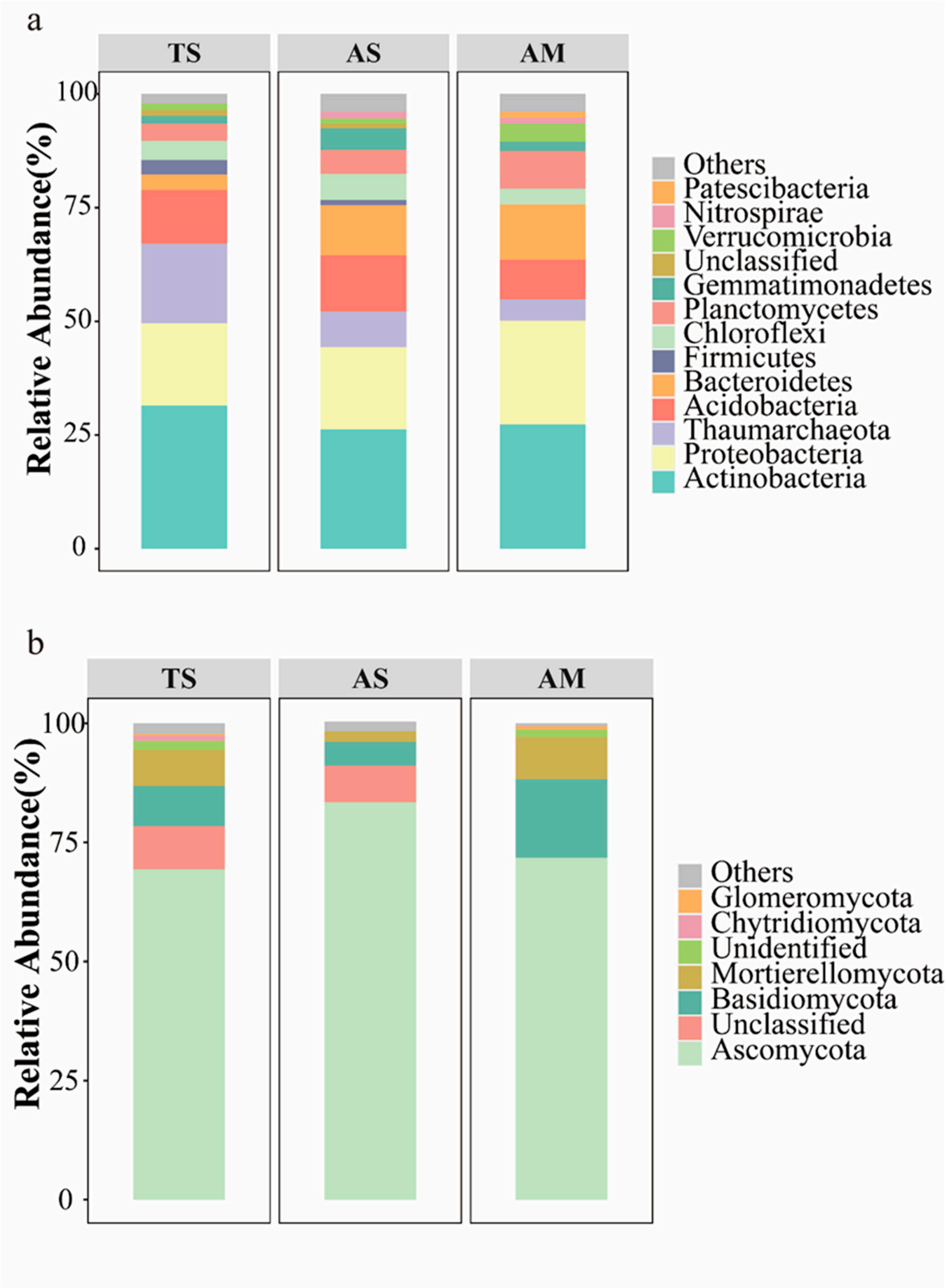

Figure S3. Relative abundance of Prokaryotic (a) and Fungal (b) communities at the phylum level across different grassland types. TS: temperate steppe; AS: alpine steppe; AM: alpine meadow.

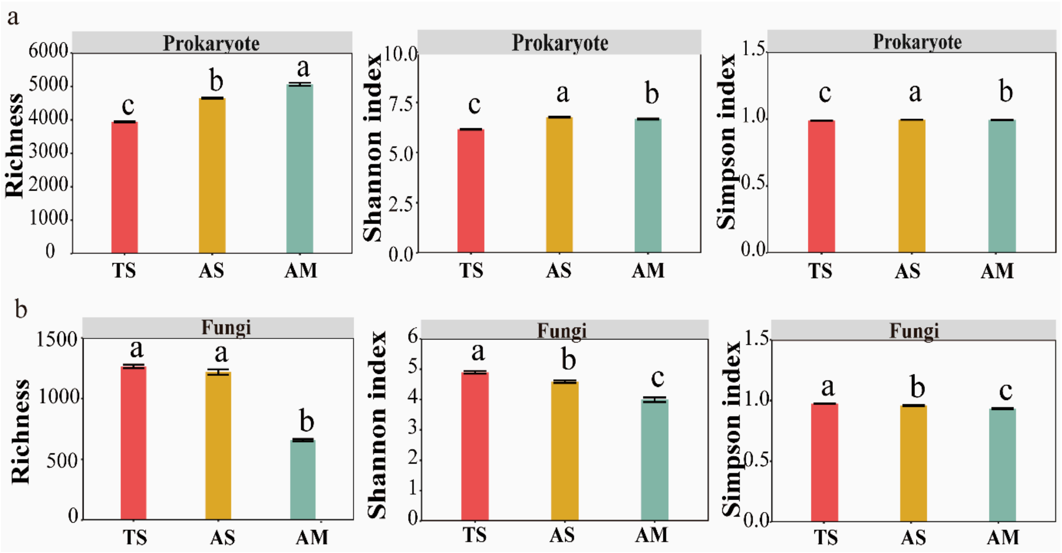

Figure S4. The alpha diversity of microbial communities across different grassland types. (a): Prokaryote; (b): Fungi. Different letters indicate significant differences among grassland types ($P \leq 0.05$). Values represent mean ± standard error. TS: temperate steppe; AS: alpine steppe; AM: alpine meadow.

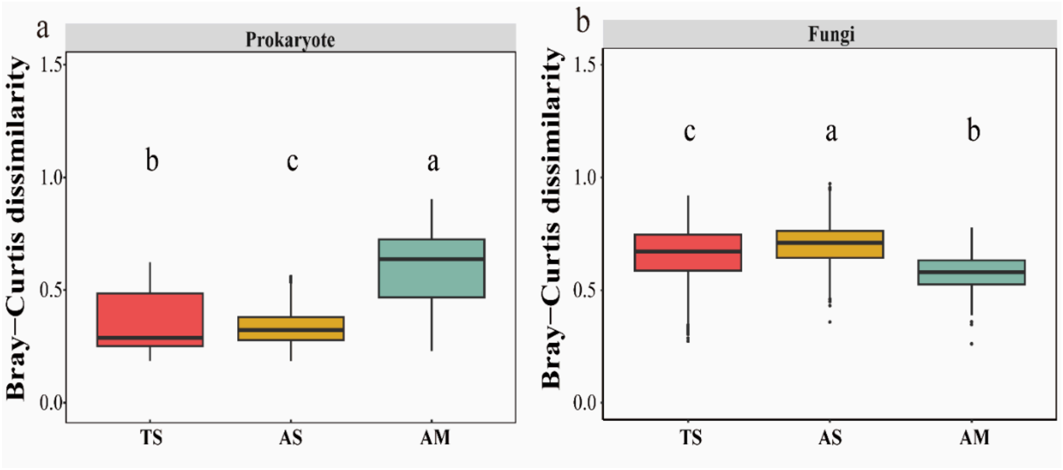

Figure S5. The beta diversity of microbial communities across different grassland types. (a): Prokaryote; (b): Fungi. Different letters indicate significant differences among grassland types ($P \leq 0.05$). TS: temperate steppe; AS: alpine steppe; AM: alpine meadow.

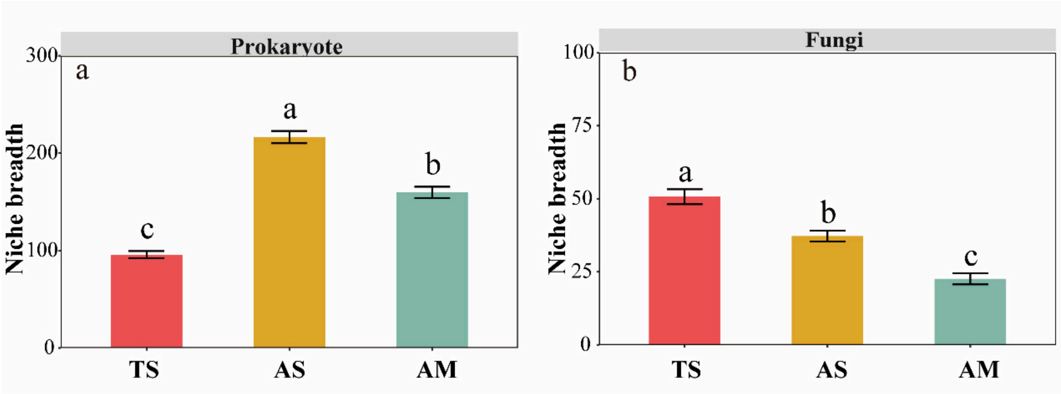

Figure S6. Niche breadth of microbial communities across different grassland types. (a): Prokaryote; (b): Fungi. Different letters indicate significant differences among grassland types ($P \leq 0.05$). TS: temperate steppe; AS: alpine steppe; AM: alpine meadow.

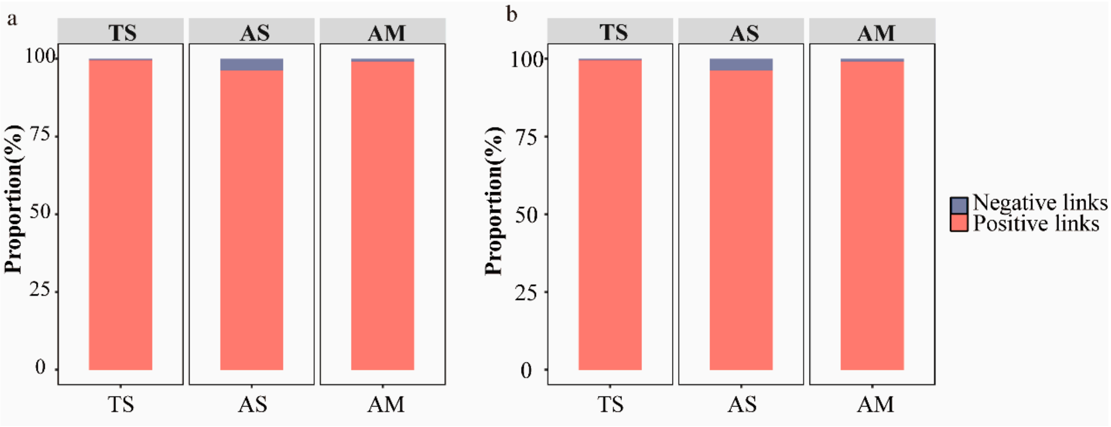

Figure S7. Relative share of positive and negative links in co-occurring networks across different grassland types. (a): Prokaryote; (b): Fungi. TS: temperate steppe; AS: alpine steppe; AM: alpine meadow.

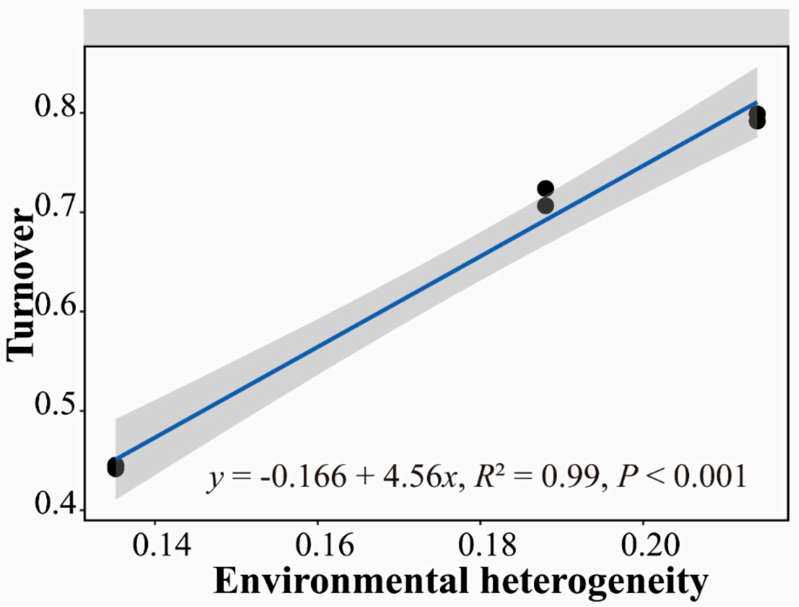

Figure S8. Relationship between environmental heterogeneity and microbial spatial turnover rate.

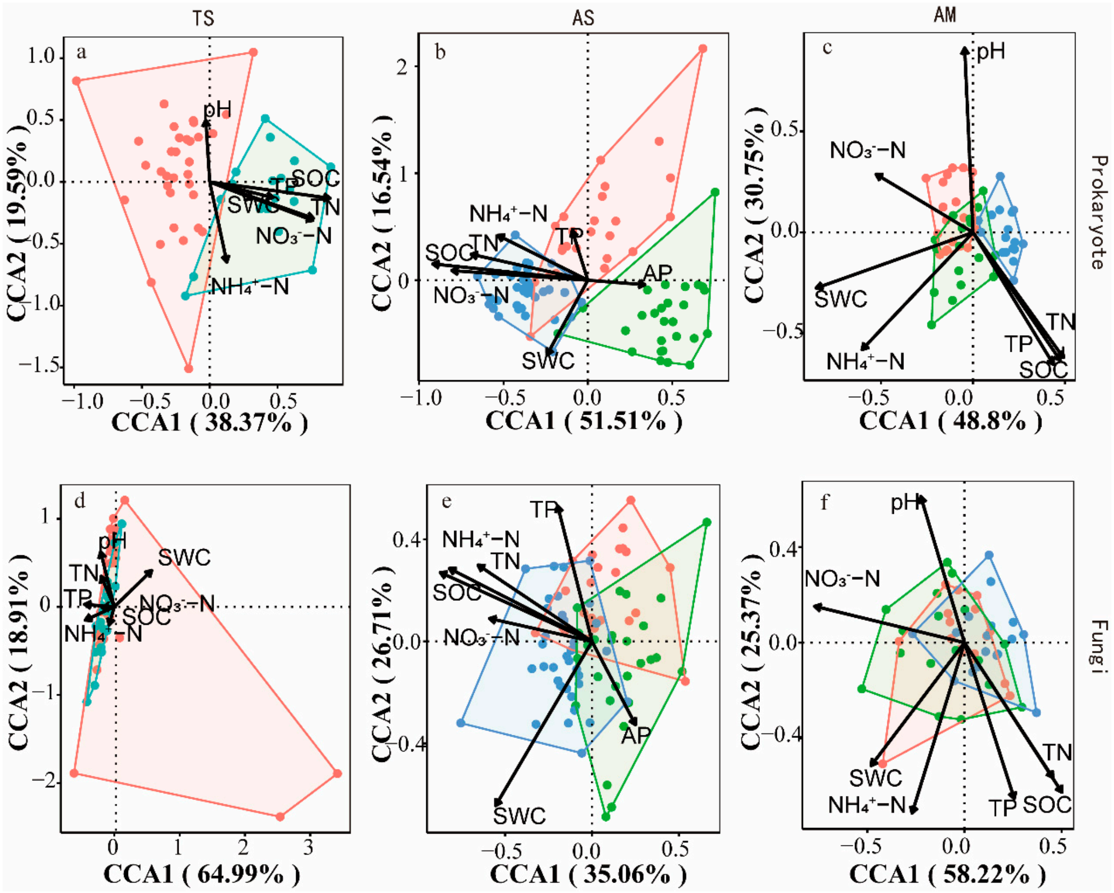

Figure S9. Canonical correspondence analysis between soil microbial communities and environmental factors across different grassland types. (a)-(c): Prokaryote; (d)-(f): Fungi. TS: temperate steppe; AS: alpine steppe; AM: alpine meadow.
